# Online sonification improves cycling performance through kinematic and muscular reorganisations

**DOI:** 10.1038/s41598-020-76498-0

**Published:** 2020-12-01

**Authors:** Benjamin O’Brien, Romain Hardouin, Guillaume Rao, Denis Bertin, Christophe Bourdin

**Affiliations:** Aix-Marseille Univ, CNRS, ISM, Marseille, France

**Keywords:** Motor control, Sensorimotor processing, Sensory processing

## Abstract

Based on a previous study that demonstrated the beneficial effects of sonification on cycling performance, this study investigated which kinematic and muscular activities were changed to pedal effectively. An online error-based sonification strategy was developed, such that, when negative torque was applied to the pedal, a *squeak* sound was produced in real-time in the corresponding headphone. Participants completed four 6-min cycling trials with resistance values associated with their first ventilatory threshold. Different auditory display conditions were used for each trial (Silent, Right, Left, Stereo), where sonification was only presented for 20 s at the start of minutes 1, 2, 3, and 4. Joint kinematics and right leg muscular activities of 10 muscles were simultaneously recorded. Our results showed participants were more effective at pedalling when presented sonification, which was consistent with previously reported findings. In comparison to the Silent condition, sonification significantly limited ankle and knee joint ranges of motion and reduced muscular activations. These findings suggest performance-based sonification significantly affected participants to reduce the complexity of the task by altering the coordination of the degrees of freedom. By making these significant changes to their patterns, participants improved their cycling performance despite lowering joint ranges of motion and muscular activations.

## Introduction

Cycling requires continuous and coordinated movements across distances over time. The pedal stroke gesture is composed of the pushing and pulling phases and high and low transitions, and most cyclists identify the pulling and transition phases as more difficult. Due to the 180$^{\circ }$ phase-offset between pedals, when pushing with one pedal, cyclists must pull with the other and thus, to be competitive, they must develop efficient pedalling techniques. Significant research has been dedicated to evaluating the efficiency of pedalling methods, which includes measuring the distribution of total forces applied to the pedal^1^ and the ratio of tangential force to total force applied to the crank^2,3^, also commonly known as *torque effectiveness*. A comprehensive review of force or torque effectiveness studies addressed constraints, such as workload and cadence, and identified interactions between cycle training and performance^4^. Although dependencies varying from physiology and nutrition to bike design can influence performance, training enables cyclists to address limitations and make adjustments to become more effective at pedalling.

Recently, a popular area of research has focused on studying the effects of augmented reality and multi-sensory feedback on sports training^5–7^. A study showed that by visualising their mechanical effectiveness per cycle, cyclists were able to orient the forces applied to the crank in a manner that was mostly tangential to the crank, and thus they improved their torque effectiveness^8^. However, given the visual demands required to be successful in real-world cycling competitions, this type of feedback appears highly incompatible with practice. Thus, in order to study the effects of augmented reality during cycling training sessions, it was important to develop a method that delivered concurrent, or “online,” feedback to participants that was based on and did not limit their performance or safety.

An alternative to visual feedback is auditory feedback. Beyond the domain of sports, several studies have shown auditory rhythmic stimuli primed participants while completing simple rhythmic exercises^9,10^. Several reviews of audio-based interventions in sports have provided neurophysiological findings that support evidence of interactions between auditory and motor systems^7,11,12^. Two sports that are similar to cycling in terms of the repetition of precisely coordinated movements are rowing and golf, and both have been subjects of sonification studies. Two major sonification studies have shown rowers were able to use online sonification to enhance boat’s forward motion^13^ and increase average boat velocity^14^. Auditory modeling and feedback have also been used in golf studies, as one study showed participants were able to perceive their golf swings from the sounds of others^15^, whereas several other studies found sonification enhanced putting performance^16^ and reduced swing variability in novices^17^.

Several pilot studies have shown cyclists were able to use sonification to enhance pedal performance^18,19^. More recently, findings have reported novice and expert cyclists were able to improve their average torque effectiveness when presented online sonification that was based on errors of performance^20,21^. These results suggest sonification can play an influential role on pedal performance, but questions remain as to which kinematic and muscular activities involved are affected and whether these changes are physiologically or biomechanically costly. Although online sonification significantly improved torque effectiveness, we reported an approximate 3% decrease in torque effectiveness for expert cyclists during 8-min training sessions^21^, which suggested participants had difficulties maintaining this improved performance over time. Thus, the goal of this study centred on identifying which structural changes were induced by sonification to improve pedal performance via torque effectiveness. To do so, we selected to examine the kinematic and muscular activities that contribute to the pedal gesture.

Numerous studies have developed methods to calculate the kinematic patterns of rigid bodies and used them to analyse changes made to the pedal stroke. Fatigue has been shown to affect ankle, knee, and hip joint movements performed while pedalling. The ankle joint showed a decrease in average angle and an increase in range of motion (RoM) during the pedalling cycle following fatigue, while the hip joint showed opposite results with a 3$^{\circ }$ reduction in RoM after fatigue^22^. Disturbing the natural pedalling cadence by ± 20% has also been shown to cause significant changes to knee and ankle movements a reduction in ankle joint RoM with the increasing cadence^23^.

*Electromyography* has been widely used in studies to measure how neural commands adapt to environmental and task demands. To gain insight into the neuromuscular strategies of lower limb movements during cycling, several studies have recorded electrical muscle activation (EMG) signals to measure changes to the lower limb muscle activations^24–26^. Researchers and trainers alike have identified these muscles as important contributors throughout the pedal phases and transitions. As our previous work showed cyclists were able to use sonification to improve pedalling performance, we wanted to use electromyography to measure and identify which muscles during which pedalling phases were affected by the presence of sonification.

In order to observe any effects of sonification on kinematic and muscular activities, it was important that participants maintained their natural pedal cadence over the course of multiple endurance trials. A principle factor in cycling endurance is the ratio of power output to energy expenditure (“gross efficiency”). Findings suggest a strong relationship between endurance optimisation and gross efficiency^27^. However, there is still much to debate as to how to adequately measure gross efficiency^28^, given cycling invariants, such as road and mountain bike cyclists or training sessions and competitions. For example, “zero load” (0 W) cycling has been shown to not provide an adequate reference for scaling work intensities^27^. However, a comparative review of methods suggested that the procedure of periodically increasing resistance was a proven method for assessing their first (VT$_{1}$) and second (VT$_{2}$) ventilatory thresholds, which can then be used for endurance training^29^. By selecting and fixing the resistances associated with their VT$_{1}$, participants would be tasked to pedal in a manner fit for endurance training, which, by maintaining their pedal cadences, would allow us to observe any changes to their kinematic and muscular activities when they were presented sonification.

Given the reported positive benefits of sonification on global torque effectiveness^20,21^, the goal of our study was to measure and identify which kinematic and muscular factors involved in the pedal stroke were affected by the presence of sonification. It was expected that the muscles responsible for knee joint flexion and hip joint extension would exhibit higher activations when participants were presented with sonification. This was based on observations that the negative torque applied to the pedal during the upward phase was mainly due to the weight of the lower limbs on the pedal. By presenting participants with sonification, we hypothesised that the presence of supplementary sensory information would create an index for them to focus on and, subsequently, lead them to stimulate and increase muscle activations in their hip and knee joints in efforts to reduce the resistance associated with the weight of their lower limbs.

## Methods

### Participants

Eight club licensed cyclists (all male) participated in our study and their details are in Table 1. All participants had a minimum of 3 years of experience cycling and cycled daily (a minimum of 10 h per week), which included sessions on stationary-bikes. All participants self-reported normal hearing. All participants were informed of their right to withdraw at any time. Informed consent was provided by all participants and, if under the age of 18, from their legal guardian(s). This study was performed in accordance with the ethical standards of the Declaration of Helsinki^30^. The Ethics Committee of Aix-Marseille University approved the protocol.

**Table 1:** Participant data.

Participant	Age	Weight (kg)	Height (cm)	VT$_{1}$ (W)	Resistance (W)	Preferred pedal cadence (Mean ± SD RPM)
1	19	72	184	300	210	87.77 ± 2.31
2	16	67	178	250	175	85.06 ± 6.8
3	18	66	187	225	160	83.62 ± 4.13
4	22	63	173	230	160	84.49 ± 1.92
5	21	74	182	175	125	90.31 ± 2.26
6	21	69	174	180	125	79.83 ± 2.09
7	18	73	174	220	155	92.63 ± 4.23
8	19	63	178	280	195	74.45 ± 4.36

### Protocol

Participants completed two cycling sessions (minimum 24 h between each session). The purpose of the first session was to identify their VT$_{1}$. Once identified, the corresponding resistance was used throughout the second session. Participants performed a 60 s - 30 W incremental cycle ergometer test to detect their VT$_{1}$^29^. Table 1 shows the corresponding VT$_{1}$ power values and resistances for each participant.

For the second session, participants performed four 6-min trials. For each trial, they were presented resistance based on their VT$_{1}$ (throughout the course of the trial) and a different auditory condition: no sonification (Silent), right pedal only (Right), left pedal only (Left), and both pedals (Stereo) sonification. The auditory condition for each trial was randomly selected (non-repeating). Each 6-min trial was bookended by a minute with no auditory feedback. The remaining 4 min were divided into alternating 20 s of the session-assigned auditory condition followed by 40 s of no auditory feedback. Participants were asked to maintain their preferred pedal cadence across the trials (see Table 1 for participant average pedal cadence). Participants were given 5 min to relax between trials. After completing the four 6-min trials, a maximal voluntary contraction (MVC) was recorded during a short sprint with a much higher resistance (approximately 500 W)^24^. The aim of calculating the MVC was to normalise EMG signals in data post-processing.

### Experimental setup

For the first session, participants used an Excalibur Sport stationary bicycle (LODE, Netherlands) and wore an analyser gases system (Quark CPET, Cosmed, Italy), which was used to identify their VT$_{1}$. For the second session, participants used the road bicycle (Merida®) and the HomeTrainer Tacx Flux.

The Rotorbike 2InPower crank, a dual-sided power meter, was used to measure the torque applied to each pedal independently (50 Hz), which was transmitted to a computer via ANT+ transmission. Depending on the session-assigned auditory condition, when negative torque values were registered, a *squeak* sound was produced in Max/MSP (Cycling ‘74, USA) and online delivered to participants via AKG K702 headphones^20,21^. The duration of the squeak was proportional to the duration of the negative torque period.

Forty-three markers (size: 19 mm; weight: 2.5 g) for a three-dimensional kinematic motion analysis were placed on the participant’s body, limbs, and head^31^. Kinematic data were captured at 200 Hz using a ten-camera Qualisys motion analysis system. Before each data collection session, a calibration of the system was performed. Only calibrations that produced average residuals of less than 0.8 mm for each camera were accepted prior to data collection.

EMG data were recorded with a Delsys Trigno system (sampling rate: 1926 Hz). 10 electrodes were placed on the lower limb following the SENIAM recommendations^32^, and taped to the skin to minimize movement artefacts. The following lower limb muscles were recorded: rectus femoris (RF), vastus lateralis and medialis (VL and VM), semi-tendinous (ST), biceps femoris (BF), gluteal (GLUT), tibialis anterior (TA), gastrocnemius lateralis and medialis (GL and GM) and the soleus (SO). Before the recording, the skin surface was shaved and cleaned to reduce skin impedance.

### Data analysis

Measurements recorded when the session-assigned auditory conditions were presented to participants were extracted and used for analysis. Torque signals were sampled at regular intervals (50 Hz), whereas pedal angles were sampled at lower interval (5 Hz). In order to calculate the completion of a cycle, the torque signals were used to interpolate and estimated pedal angles (0.5$^{\circ }$ steps) via the MATLAB cubic interpolation function *interp1* (MATLAB 2016b, MathWorks Inc, USA). The performance of each cycle was assessed by calculating the torque effectiveness (TE), where $\tau ^+$ the total positive torque over the cycle and $\tau ^-$ the total negative torque over the cycle (Eq. 1)^20,21^. TE was considered 100% if there was no negative torque during the cycle.

1 $$\begin{aligned} TE = 100 * \frac{\tau ^+ +  \tau ^-}{\tau ^+} \end{aligned}$$

Marker data were filtered using a Butterworth 6th order low-pass filter (cut-off frequency: 15 Hz). The kinematics at hip, knee and ankle joints of the right lower limb were computed in the sagittal plane using Visual3D (v6 Professional, C-Motion, USA). Ankle, knee, and hip joint angles were calculated over each cycle. The crank angle was also calculated from the retro-reflective markers placed on the crank extremities. Based upon the detection of the vertical position of the right crank, joint kinematics and EMG data were split into cycles and time-normalised to 101 points over the pedalling cycle.

To accurately measure the maximal muscle activity performed while cycling, the sprint method of normalization was used, as it has been proven to be reliable in nature to subsequent cycling trials^33^. Thus, the EMG signals recorded during the short sprint that followed the four 6-min trials were used for normalization. The EMG signals were filtered with a Butterworth 2nd order band-pass filter (frequency window: 20–400 Hz), rectified, and then low pass filtered (Lag time: 0; order: 2; cut-off frequency: 10 Hz) using MATLAB self-developed routines.

### Statistical comparisons

To analyse the percentage of torque effectiveness, we opted for a sample size that reflected ones used in similar studies^27,34,35^. In addition, we computed *a priori* power analysis for within-between participants Repeated Measures (RM) ANOVA (medium effect-size = 0.5, $\alpha$ = 0.05), which revealed that a sample size of at least 8 was sufficient to reach a power of 0.95. RM ANOVA was conducted with auditory condition and pedal factors. Where main effects and interactions were detected, post-hoc Bonferroni-adjusted t-tests were carried out. Significant findings were reported XX ± YY (XX: mean; YY: s.d) and accompanied by *p*-values. Where the assumption of sphericity was violated, Greenhouse-Geisser adjustments are reported.

Right leg-only kinematics and EMG analyses were performed with the statistical MATLAB package SPM1D (version M.0.4.5)^36^. Statistical Parametric Mapping (SPM) was developed initially in neuroimaging in the mid-1990s, but has more recently been applied in the field of biomechanics. This method looks at the probabilistic inferences regarding experimental data based on mean, standard deviation, and sample size data, where the start of the pedal stroke (Time 0%) was identified when the right crank was positioned at the top (0$^{\circ }$). To estimate 1D variance, several model variables were required for an SPM analysis^36^, however, these were unavailable due to the novel but recognized uses of SPM to analyze kinematic and EMG signals in cycling. Paired Hotelling’s $T^{2}$ tests were used to evaluate the main effects of auditory condition factors on the multidimensional lower limb joint angles and EMG waveforms during pedalling. This multidimensional analysis is usually called vector field analysis. Where main effects and interactions were detected on the vector field analysis, post-hoc Bonferroni-adjusted t-tests were carried out on the 1D variables between the experimental conditions during the cycle period where the vector field reported significance ($\alpha$ = 0.017). This latter analysis acted as a proxy for post-hoc testing on the 1D variables that significantly contributed to the vector field differences.

## Results

### Torque effectiveness

For torque effectiveness (TE) we found a main effect on auditory condition F$_{3,21}$ = 9.65, $p<$ 0.001, $\eta _{p}^{2}$ = 0.58, but no significance on pedal, $p>$ 0.05. Post-hoc Tukey tests showed participants were significantly more effective at pedalling when presented the Stereo condition (95.57 ± 1.65%) when compared to auditory conditions Silent (86.56 ± 2.01%), $p<$ 0.01, and Left (92.48 ± 1.99%) conditions, $p<$ 0.05. There were no significant differences in torque effectiveness between the Stereo and Right (94.32 ± 2.01%) conditions, $p>$ 0.05.

**Figure 1:**
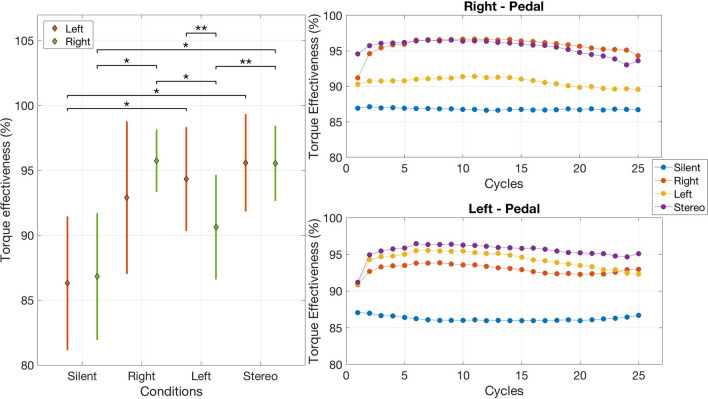
Left: Average torque effectiveness percentage over all the participants across auditory conditions for both pedals. {*, **} represent $p<$ {0.05, 0.01} significance between means with CI set to 95%. Vertical lines represent s.e.m; Right: Average torque effectiveness percentage per cycle during each auditory condition per pedal.

We found an interaction between auditory condition * pedal F$_{3,21}$ = 5.14, $p<$ 0.01, $\eta _{p}^{2}$ = 0.42. Analysing each pedal between conditions, post-hoc Tukey tests showed that participants were significantly more effective at pedalling with their left pedal when presented the Left (94.33 ± 2.04%) and Stereo (95.55 ± 1.48%) conditions when compared to the Silent condition (86.3 ± 2.63%), $p<$ 0.05. Similarly the post-hoc Tukey tests similarly revealed participants were more effective at pedalling with their right pedal when presented the Right (95.75 ± 1.23%) and Stereo (95.55 ± 1.91%) conditions when compared to the Silent condition (86.83 ± 2.49), $p<$ 0.05. Participants were less effective at pedalling with their right pedal when presented the Left condition (92.89 ± 2.99%) in comparison to the Right condition, $p<$ 0.05, and the Stereo condition, $p<$ 0.01. Finally, by analysing each condition between pedals, post-hoc tests revealed that when participants were presented the Left condition, they were significantly more effective at pedalling with their left pedal when compared to their right, $p<$ 0.01. Figure 1 illustrates these significant findings (**Left**) and participant average torque effectiveness per cycle over the four auditory conditions for each pedal (**Right-Top**: right pedal; **Right-Bottom**: left pedal).

### Joint kinematics

SPM vector field analysis found significant main effects between all auditory conditions for joint kinematic coordination (Table 2). Our findings show sonification affected the pedalling pattern over the course of the cycle completion.

**Table 2:** Paired Hotelling’s $T^2$ test results for kinematic activity.

Condition 1	Condition 2	$\mathbf{T} ^{2}$ (%)	*p*	Cycle completion (%)
Silent	Left	19.38	**	1–16
			***	68–100
Silent	Right	19.64	***	1–4
Silent	Stereo	17.49	***	1–62
			**	66–88
			*	97–100
Left	Right	17.92	**	19–25
			***	30–54
Left	Stereo	21.06	***	1–61
			**	91–100
Right	Stereo	17.81	**	73–87

Where {*,**,***} mark significance for $p<$ {0.05, 0.01, 0.001}.

**Figure 2:**
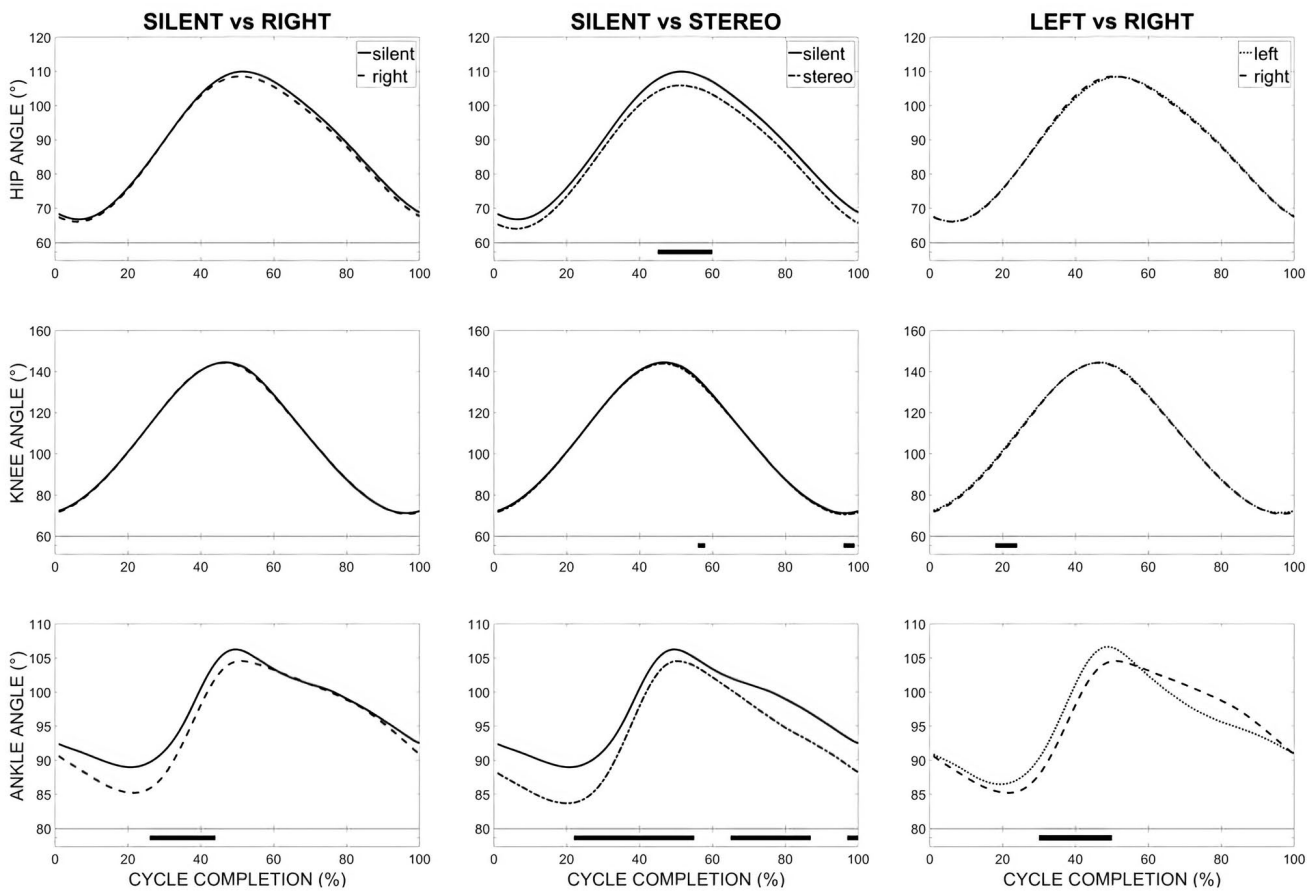
SPM *t* test analysis of kinematic activities between conditions over course of cycle completions for ankle, knee, and hip joints. Standard deviations are not presented for more clarity. Significant main effects are highlighted (black horizontal bars at the bottom of the figure) during corresponding time periods.

Post-hoc Tukey tests revealed significant findings for ankle, knee, and hip joint kinematics (Table 3). In general, when compared to the Silent condition, participants significantly reduced the absolute angles and ranges of motion of their ankle and hip joints when presented sonification, however, with specificity to the conditions (Fig. 2). While the Stereo condition significantly affected performance throughout almost the entirety of cycle (from 1 to 62%, 66 to 88%, and 97 to 100%), the Left and Right conditions had inverse effects: the Left condition influenced performance from the push-on (from 0 to 15%) and pull phases (from 67 to 100%), which is around dead centre at the top, whereas with the Right condition influenced from the push-off to the pull-on phrases (25 to 61%), dead centre at the bottom. In addition, when compared to the Silent condition, participants decreased their hip angle excursion by 4o (from 1 to 62%) when presented the Stereo condition, $p<$ 0.05.

**Table 3:** Post-hoc Tukey Test results for kinematic activity.

Condition 1	Condition 2	Cycle completion (%)	Ankle		Knee	
			$\text {t}_{\text {crit}} (\%)$	*p*	$\text {t}_{\text {crit}} (\%)$	*p*
Silent	Left	68–100	− 3.6	***		
Silent	Right	26–62	− 4.7	***		
Silent	Stereo	1–62	− 6.1	***	− 0.9	*
		66–88	− 4	**		
		97–100	− 4.2	*	− 0.06	**
Left	Right	19–25			− 0.08	**
		30–54	− 2.7	**		
Left	Stereo	1–61	− 2.8	***	− 0.8	***
		91–100			− 0.7	***
Right	Stereo	73–87	− 4.1	***	− 0.5	***

Where {*,**,***} mark significance for $p<$ {0.05, 0.01, 0.001}.

### Muscle activity

SPM vector field analysis revealed significant differences between all auditory conditions for muscular activities with the exception of the Left and Right conditions comparison (Table 4). The largest difference observed was between the Silent-Stereo and Silent-Right conditions.

**Table 4:** Paired Hotelling’s $T^2$ test results for muscle activity.

Condition 1	Condition 2	$\text {T}^{\text {2}}$ (%)	*p*	Cycle completion (%)
Silent	Left	73.17	**	1–4
			**	28–31
			*	54–55
			***	88–100
Silent	Right	73.72	*	1–4
			***	13–48
			*	51–52
			**	53–60
			*	79–80
			***	88–100
Silent	Stereo	73.94	*	1–2
			***	14–44
			***	87–94
			**	95–100
Left	Right	72.57		
Left	Stereo	73.19	*	1–3
			**	23–28
			*	35–37
			***	88–100
Right	Stereo	74.85	***	9–15
			*	96–97

Where {*,**,***} mark significance for $p<$ {0.05, 0.01, 0.001}.

**Figure 3:**
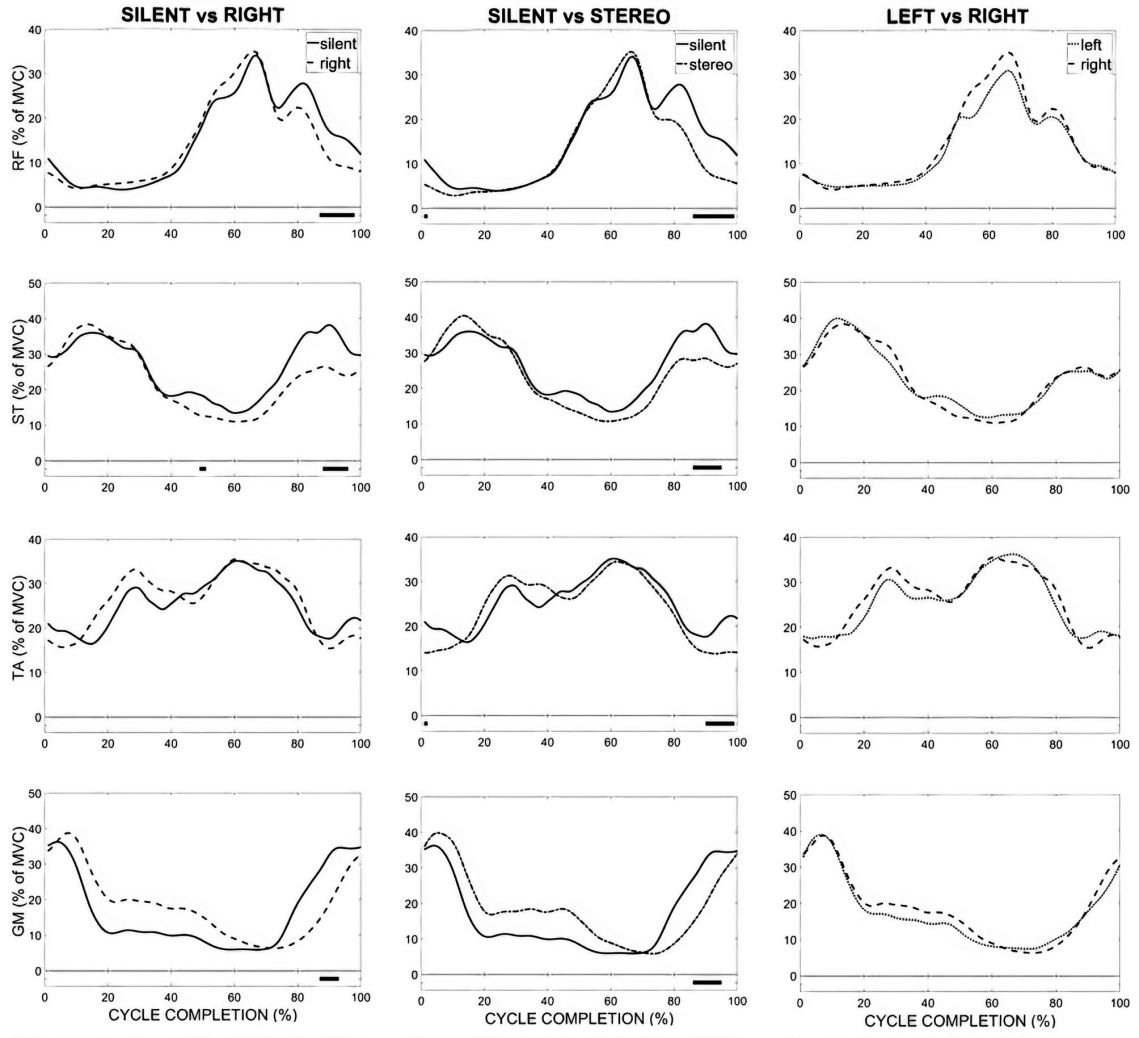
SPM *t* test analysis of muscular activities between conditions over course of cycle completions for RF, ST, TA, and GM muscles (top to bottom). Standard deviations are not presented for more clarity. Significant main effects are highlighted (black horizontal bars at the bottom of the figure) during corresponding time periods.

Post-hoc Tukey tests revealed major findings during the push-on (from 0 to 15% of the pedalling cycle) and pull-off phases (from 85 to 100%) for muscles RF, ST, TA, GL, and GM (Table 5). In general, the Silent condition showed higher muscle activations than all other auditory conditions during the pull-off phase (from 86 to 100%) and during the push-off phase (from 12 to 47%) as seen in Fig. 3. In addition, when compared to the Silent condition, participants decreased their activations for the BF muscle by 6% (from 88 to 100%) when presented the Right condition, $p<$ 0.01.

**Table 5:** Post-hoc Tukey test results for muscle activity.

Condition 1	Condition 2	Cycle completion (%)	RF		ST		TA		GL		GM	
			$\text {t}_{\text {crit}}$ (%)	*p*	$\text {t}_{\text {crit}}$ (%)	*p*	$\text {t}_{\text {crit}}$ (%)	*p*	$\text {t}_{crit}$ (%)	*p*	$\text {t}_{crit}$ (%)	*p*
Silent	Left	1–4	− 2.5	*								
		88–100	− 6	***	− 12	***						
Silent	Right	79–80			− 9.5	***					− 11	**
		88–100	− 6.5	**	− 10.5	**			− 6	**	− 13.5	*
Silent	Stereo	1–2	− 5	***								
		87–94	− 8	***	− 10	**	− 4	*	− 6.5	**	− 12.5	***
		95–100	− 8	***	− 4	*	− 8	***			− 3	**
Left	Stereo	1–3	− 2	*			− 3.5	***				
		88–100	− 2.5	**			− 5	***				
Right	Stereo	9–15	− 1	***			− 3	*				
		96–97	− 2	***			− 4	*				

Where {*,**,***} mark significance for $p<$ {0.05, 0.01, 0.001}.

## Discussion

### Torque effectiveness

Our results support previously reported findings of the effectiveness of sonification on the pedal stroke^20,21^. Participants were less effective at pulling when presented sonification on their opposite pedal. This point was underscored by our significant findings, as participants were 3.71 ± 0.9% more effective with their left pedal as opposed to their right when presented Left sonification. These observations suggest the importance of measuring the muscle and kinematic activities that contribute to bi-lateral pedalling. A major take-away was that the presence of any sonification—uni- or bi-lateral—enhanced performance, as participants applied less negative- and positive-torque. This decrease in positive torque can be understood as a product of the effects of sonification on joint kinematics and subsequent lower limb muscle activities, which are described in greater detail below.

The initial spike in torque effectiveness between cycles 1 and 2 as illustrated in Fig. 1 (**Right-Top** and **Bottom**) suggests the near-immediate effect of sonification on pedal performance. As evidenced by the absence of a spike and the overall lower percentage of effectiveness during the Silent condition, participants had no cue or indication of their performance. With the presence of sonification, in general, participants were able to sustain their pedalling effectiveness. We designed our study so that, if selected, sonification would not be presented throughout, but rather repeated with short durations (20 s bouts). Our findings suggest that this approach worked, however, a future study might examine the frequency and duration of presenting sonification and its lasting effects on pedal performance. In any event, these observations highlight how error-based auditory feedback can play a significant role in alerting movement performance. Moreover, given the cycling experience of our participants, the findings further support the idea that online sonification can be an effective tool for improving pedal performance.

### Joint kinematics

Our kinematic analysis suggests sonification seemed to affect the entire pedalling cycle, however, principally in the ankle and knee joints. With the exception of the 4$^{\circ }$ decrease in the hip excursion during the push phase and the start of the pull when comparing the Silent and Stereo conditions, sonification had no effect on the hip joint. The pronounced decrease of absolute values and amplitudes on the ankle joint suggests sonification affected it first. The kinematic data suggests participants “locked” their ankles to better control the direction of forces applied to the crank. This observation was clearly visible when the pedal was positioned at the bottom dead centre—the end of the push and start of the pull phase.

This kinematic reorganisation helped to control and direct the external forces applied on the pedal by “removing” an additional degree of freedom that was not suitable to orient the resultant pedal force in an efficient direction, which was backwards and backwards-upwards at the start of the pulling phase. As a result, the cyclists improved their torque effectiveness by reducing the negative torque applied to the pedals. These results fall in line with the existing body of literature showing that more distal joints are typically more affected when an intervention is done either on the sport equipment^37,38^ or the environment^39^. With less movement in their ankles, participants were not required to make large angular movements at the knee and hip joints, which explains the reduced angular amplitudes at these joints. Thus, sonification had a blocking effect on the ankle joint, as it reduced the number of degrees of freedom available for mobilization and helped to direct pedal forces to the back- and up-wards.

While there is clear evidence that sonification can be used to improve torque effectiveness, its effects—short- and long-term—on reducing ankle, knee and hip joints mobility demand further study. Indeed, the locking of the ankle joint and the subsequent limitation in knee and hip joint excursions drastically changed the pedalling pattern.

### Muscle activity

Compared to the Silent condition, sonification appeared to decrease activations for the RF/ST and GM-GL/TA muscles. With regards to the pedal stroke gesture, the RF contributes to the hip joint flexion and knee joint extension, whereas the ST muscle has an opposite action by extending the hip joint and flexing the knee joint. In addition, GM, GL, and TA muscles are involved in the plantar (GM and GL) and dorsal flexion (TA) of the ankle joint.

As discussed in greater detail above, sonification caused a significant amplitude reduction in the ankle, knee, and hip joint kinematics. With more limited ranges of motion, participants were not required to activate as much of the lower limb muscles mobilizing the three major joints of the lower limb. Of particular interest, this result opposed our expectations of observing increased activations of the knee joint flexors and hip extensors, which indicates that even trained cyclists have the capacity to increase their TE.

The changes in the muscular pedalling pattern induced by the presence of sonification once again highlight the high complexity of the pedalling task in terms of muscle coordination. Indeed, muscular coordination was strongly affected, as participants tried to minimize or remove negative torque applied to the sonified crank side as well as decrease the positive torque contribution. Our analyses of the muscles with modified activations showed that the muscular reorganisation affected mainly the bi-articular muscles. These muscles have unique mechanical and functional properties, as they are known to be efficient in controlling the direction of external forces due to their paths spanning several joints^40,41^. Hence, although the muscular coordinations were deeply modified, these modifications specifically targeted muscles that were both available and capable of strongly influencing the direction of external forces on the whole kinematic and muscular chains of the lower limb. The influence of sonification on muscle coordination goes against previous studies that have reported rather consistent pedalling patterns among different mechanical constraints^42^ and could reveal that providing online auditory feedback via sonification is an efficient way of inducing drastic changes in already established coordination patterns, and thus ultimately improve cycling performance.

Such a reduction in muscle activations contributed to an increase in torque effectiveness when presented sonification, which is relevant to improving pedal performance. While this is certainly of interest, it has to be balanced with the large changes observed in joint kinematics and the associated potential increase in exertion and decrease comfort, which, if performed too much or without advisement, could potentially lead to injuries. Underscoring this latter point, participants were not only affected as expected, but the presence of sonification led to deep muscular and kinematic reorganisation. In this case, the purpose of sonification was to present participants a real-time index based on their performance errors. We designed our study, so that sonification was not presented to them throughout the entirety of the 6-min trials, but rather four times per trial with a maximum duration of 20 s and only if negative torqued was measured during this period. Thus, it was possible that participants became more sensitive to the presence of artificial auditory information and subsequently more reactive in terms of their muscle movements. This result suggests that the frequency of presenting sonification plays an important role in changing the muscular patterns associated with the pedal stroke.

### Combined effects

Torque effectiveness was significantly higher when participants were presented sonification, which is an important point in terms of motor output. Of course, this observed effectiveness was derived from changes to the biomechanical pedalling patterns made by participants. The joint ranges of motion were reduced, especially at the ankle and knee joints. In particular, when participants were presented Stereo condition, we identified the combination of cross effects on the ankle and hip joints. Participants were asked to not only complete a complex task—maintain pedal cadence with a resistance that corresponded to their VT$_{1}$)—but were also (occasionally) provided auditory feedback based on performance. Their muscular and kinematic data suggests they did their best to minimise the degrees of freedom by making significant changes to their patterns, which resulted in less joint excursions and muscular activations. However, this decrease in amplitude moved them away from their usual pedal pattern, which, in turn, made it (appear) harder and more tiring. By reducing the amplitude of their movements and the positive torque, participants also reduced their need to activate their muscles, which might explain their increase in torque efficiency.

The effects of sonification on muscle activity were systematic and localized during the pedalling cycle. Because the electrodes were placed on the right leg, we observed almost identical localization of the effect of the sonification between the Right and Stereo conditions, where there were significant differences with the Silent condition and little differences between them. Interestingly, our findings suggest a cross effect with the Left condition, as we observed that, when compared to the Silent condition, it produced significant effects on the right ankle and knee joints during the start of the push and end of the pull phase. These effects were inversional or anti-phasal to the Right and Stereo conditions when they were compared to the Silent condition. Although these findings appear to be a product of the mechanical coupling of the cranks, an important finding was that the Stereo condition produced even more significant changes, which revealed, once again, the high complexity of the task and the high potential of the sonification to affect pedalling patterns.

## Conclusion

This study supports previous findings on the effects of sonification on sports-related performance. There is increasing evidence that presenting high-level athletes with artificial auditory information enhances performance, which, interestingly, has been observed across the sports domain, including ball-related sports^15,43,44^ and sports with continuous and repetitive movements^21,45,46^. While many studies have focused on reporting the effects of sonification on performance, our study not only found that online error-based sonification improved pedal performance, but it also identified the joints and muscles that were affected, as participants made significant reorganisations to their kinematic and muscular activities. Thus, participants improved pedal force effectiveness despite lowering their joint ranges of motion and muscle activations. Relative to competitive cycling, our results provide a step forward in isolating and pinpointing the locations affected by sonification, which present new questions regarding the relationships between the auditory perception and neuromuscular systems.

When considering the practical applications of sonification on pedal performance, it is possible sonification might be more beneficial on recurrent or short-duration exercises. It appears to cause a number of changes to the muscular and kinematic pattern of pedal stroke gesture. However, these modifications could be deleterious for expert, unsupervised, or aged cyclists, as our results suggest sonification caused neuromuscular disorganisation. As voluntarily reported by some of our participants, they felt early fatigue and slight muscle pains. This could very well have been the dual-effect of perceiving difficulty and muscular fatigue. Yet, sonification could be used in exercises designed to learn or re-learn of the pedal stroke gesture. Further research with a pool of novice or rehabilitating cyclists might offer another promising angle on the effects of sonification on pedal performance.

Today there are many mobile products available to competitive cyclists that offer performance information such as rotations per minute or work performed, as well as standard features, such as GPS. However, most of these applications are limited in delivering auditory feedback and, to the authors’ knowledge, no features offer online sonification based on errors of performance. In parallel to our study, we developed a mobile application that measured the forces applied to each pedal, using a different but commercially available pedal set, and delivered online sonification based errors of performance. Our developmental goal was to use this application to study the effects of error-based sonification in real-world cycling scenarios, including on road and mountain biking environments. By using our application, we are able to not only study the effects of sonification outside of the laboratory setting, but also focus on when participants are presented sonification relative to their position and elevation by using their GPS locations. Our findings from this study add a new dimension to consider, as we aim to integrate, for example, wearable IMUs, into our system to examine the effects of sonification on kinematics in real-world cycling situations. In addition, several error-based sonification strategies have been developed in an effort to offer users a way of personalizing their engagements with sound, much like high-level athletes have idiosyncratic ways of moving their bodies and limbs. This study advances previous research on the effects of sonification in sports by identifying the locations of joint kinematics and muscular activations in competitive cyclists.

## Data Availability

Data is available by request.
